# Investigating the Role of the Zinc Finger Protein ZC2HC1C on Autism Spectrum Disorder Susceptibility

**DOI:** 10.3390/medicina61040574

**Published:** 2025-03-24

**Authors:** Simone Treccarichi, Mirella Vinci, Antonino Musumeci, Rosanna Galati Rando, Carla Papa, Salvatore Saccone, Concetta Federico, Pinella Failla, Martino Ruggieri, Francesco Calì, Agata Polizzi, Andrea Praticò

**Affiliations:** 1 Oasi Research Institute—IRCCS, 94018 Troina, Italy; streccarichi@oasi.en.it (S.T.); mvinci@oasi.en.it (M.V.); amusumeci@oasi.en.it (A.M.); rgalati@oasi.en.it (R.G.R.); cpapa@oasi.en.it (C.P.); pfailla@oasi.en.it (P.F.); 2 Department Biological, Geological and Environmental Sciences, University of Catania, Via Androne 81, 95124 Catania, Italy; salvatore.saccone@unict.it (S.S.); concetta.federico@unict.it (C.F.); 3 Unit of Pediatric Clinic, Department of Clinical and Experimental Medicine, University of Catania, Via Santa Sofia 89, 95123 Catania, Italy; m.ruggieri@unict.it; 4 Department of Formative Process, University of Catania, Via Teatro Greco 84, 95124 Catania, Italy; agata.polizzi1@unict.it; 5 Deparment of Medicine and Surgery, University Kore of Enna, Cittadella Universitaria, 94100 Enna, Italy; andrea.pratico@unikore.it

**Keywords:** *ZC2HC1C* gene, autism spectrum disorder, zinc finger C2HC/C3H type, autosomal recessive

## Abstract

*Background and Objectives*: Zinc finger proteins are important transcription factors that regulate gene expression and play a critical role in neurodevelopment including autism spectrum disorders (ASDs). They are involved in a variety of cellular processes, including cell proliferation, differentiation, and apoptosis. *Materials and Methods*: Whole-exome sequencing (WES) analysis on a patient diagnosed with ASD. *Results*: Sequencing identified a homozygous insertion causing a stop codon, resulting in the removal of several functional domains including the zinc finger C2HC/C3H type of the ZC2HC1C protein. To date, no MIM entry has been assigned to the detected gene. In silico predictions described the variant as likely pathogenic, indicating an autosomal recessive inheritance pattern. In this study, we hypothesize that this homozygous mutation disrupts protein function and may represent a susceptibility gene for autism. The parents and the patient’s sister were healthy and carry the variant in the heterozygous condition. This gene is expressed in brain tissues showing high expression in both the choroid plexus (ChP) and midbrain, whose dysfunctions, as reported, may lead to ASD. Moreover, predictive pathway analyses indicated the probable involvement of this gene in primary cilia development. This process has been frequently linked to neurodevelopmental impairments, such as autism, as documented in previous studies. *Conclusions*: Further analyses are needed via in vitro functional assays or by *ZC2HC1C* gene knockout to validate its functional role.

## 1. Introduction

Zinc finger (ZNF) proteins represent a prominent category of proteins highly prevalent in eukaryotic genomes. They are characterized by the presence of multiple zinc finger domains, each of which possesses the remarkable ability to selectively bind to specific DNA or RNA sequences and interact with various proteins [1]. This distinctive property enables ZNF proteins to exert regulatory control over gene expression both at the transcriptional and translational levels. In the context of neurological diseases, extensive research has consistently demonstrated a significant linkage between ZNF proteins and the pathogenesis of various neurological disorders. In fact, several works associate mutations in zinc finger proteins with several diseases, encompassing epilepsy, schizophrenia, and autism [1,2].

The classic C2H2 zinc finger protein comprises two conserved cysteines and two conserved histidine residues, which, in conjunction with a zinc ion, form two β sheets and an α helix. The HUGO Gene Nomenclature Committee (HGNC) categorized non-classical zinc finger types into 30 distinct groups based on variations in the quantities of cysteines and histidines in 2015 [1,3]. It is worth noting that numerous variants within genes containing the C2H2 domain have been associated with autism [4,5,6]. Conversely, various zinc finger domains have been discovered. For instance, zinc finger proteins may exhibit atypical domains. Within this context, the C2HC/C3H type consists of three cysteine and one histidine residues [7,8,9]. Zfp474, a protein exclusively located in the testis and ovary, is believed to act as a germ cell-specific transcription factor, playing crucial roles in spermatid differentiation and oocyte development [10]. Subsequently, the homolog Zfp474 was found to show testis-specific expression in the dogfish (*Scyliorhinus canicular*) [11], and additional research indicated its expression in mouse cilia cells [12]. This rare and atypical domain was also identified in Zinc finger C2HC domain-containing protein 1C (ZC2HC1C).

This gene was previously associated with primary congenital glaucoma, although further studies are needed for validation [13]. Conversely, significant overexpression of the *ZC2HC1C* gene was observed in the mouse hippocampus as a result of perinatal exposure to glyphosate, compared to untreated controls [14]. Additionally, benign somatic mutations within *ZC2HC1C* were detected by whole-exome sequencing in subjects with primary gastric cancer [15]. Moreover, the construction of a brain region of interest (ROI) guided by an algorithm incorporating structural constraints revealed a plausible diagnostic correlation between ZC2HC1C and Alzheimer’s disease (AD), suggesting its potential as a biomarker. Notably, prediction analysis indicated a strong association of ZC2HC1C with the left hippocampus [16]. The study utilized a novel algorithm to identify brain regions most affected by multiple risk genes, emphasizing their biological significance.

A previous study unveiled a strong protein–protein interaction between ZC2HC1C and various ciliary proteins involved in both primary cilia development and intraflagellar transport [17]. Within this context, intraflagellar transport (IFT) is a bi-directional process by which particles are carried within the cilia or flagella. This process is essential for ciliary growth and functional maintenance. The IFT protein complex is involved in motor functions for anterograde and retrograde transport towards the ciliary tip [18]. Evidence suggests that dysfunction in primary cilia is implicated in ciliopathies and may contribute to the etiology of various psychiatric and developmental disorders, encompassing autism spectrum disorder (ASD), offering insight into shared molecular pathways [19,20,21,22].

Within this framework, the primary aim of the current study is to establish, for the first time, a likely association between an identified homozygous variant within *ZC2HC1C* (identified by whole-exome sequencing) and a patient affected by autism spectrum disorder (ASD).

## 2. Materials and Methods

### 2.1. Next-Generation Sequencing

The extraction of the genomic DNA was conducted from peripheral blood leukocytes obtained for both the patient and parents. DNA extraction was carried as previously described [23,24]. Whole-exome sequencing (WES) analysis was performed using the Ion AmpliSeq™ Exome RDY Kits following the manufacturer’s instructions (Thermo Fisher Scientific, Waltham, MA, USA). The quality of libraries was assessed using DNA 1000 chips on the Tape Station 4200 (Agilent, Santa Clara, CA, USA) and Qubit dsDNA BR Assay Kits (Invitrogen, Waltham, MA, USA). For the analysis, pooled libraries were utilized for the emulsion PCR on the Ion Chef Instrument, according to the manufacturer’s instructions (Thermo Fisher Scientific, Waltham, MA, USA). Finally, each loaded Ion 550™ chip on the S5 System (Thermo Fisher Scientific, Waltham, MA, USA) was sequenced. A total of 98% of the regions of interest have a minimum coverage of at least 20X. The presence of the variant was confirmed by conventional Sanger sequencing (Applied Biosystems Prism 3130 DNA Analyzer, Thermo Fisher Scientific, Waltham, MA, USA) for the patient, his healthy sister and both their parents. The flanking primers utilized for the conventional sequencing were designed through the online tool available from the National Center for Biotechnology Information (NCBI) (https://www.ncbi.nlm.nih.gov/tools/primer-blast/) (accessed on 10 June 2024).

### 2.2. Data Analysis

The amino acid sequence was retrieved and modified from UCSC Genome Browser (https://genome.ucsc.edu/) (accessed on 10 September 2024). All the common variants, non-exonic polymorphisms, were excluded, keeping polymorphisms with a minor allele frequency (MAF) of <1% in the following public databases: gnomAD Exomes v.3.1.2, 1000 Genome Project and Exome Sequencing Project (accessed on 10 September 2024). The pathogenic variants were searched on The Human Gene Mutation Database (HGMD Professional 2023, https://www.hgmd.cf.ac.uk/ac/introduction.php, accessed on 10 September 2024). VarAFT filtering (https://varaft.eu/) (accessed on 10 September 2024) on vcf files was carried out [25]. The identified variant was classified using the “American College of Medical Genetics” (ACMG) guidelines [26] and performed using VarSome in accordance with a previous study [27].

ZC2HC1C protein structure prediction was carried out using the UniProt database (https://www.uniprot.org/) (accessed on 10 September 2024) and UCSF ChimeraX software developed by the Resource for Biocomputing, Visualization, and Informatics at the University of California, San Francisco, with support from National Institutes of Health R01-GM129325 and the Office of Cyber Infrastructure and Computational Biology, National Institute of Allergy and Infectious Diseases (https://www.cgl.ucsf.edu/chimerax/) (accessed on 10 September 2024) for the high-quality protein modeling and visualization. Gene expression was analyzed from The Human Protein Atlas database (https://www.proteinatlas.org/) (accessed on 10 September 2024) and GTEx database (https://www.gtexportal.org/) (accessed on 10 September 2024).

Allele frequencies were obtained from the Genome Aggregation Database (GnomAD) (https://gnomad.broadinstitute.org/) (accessed on 10 September 2024). Subsequently, Hardy–Weinberg principle analysis was conducted to determine the frequency of the mutated allele in homozygous conditions across the population. The analysis employed the following formula, as described in previous studies [28,29]:

p^2^ + 2pq + q^2^ = 1

Here, p^2^ represents the proportion of individuals homozygous for the dominant allele ‘p’; 2pq represents the proportion of heterozygous individuals; q^2^ represents the percentage of individuals homozygous for the recessive allele ‘q’.

The evaluation of protein–protein interaction was documented in the databases STRING (https://string-db.org/) (accessed on 10 September 2024), BIOGRID (https://thebiogrid.org/) (accessed on 10 September 2024), and IntAct (https://www.ebi.ac.uk/intact/home) (accessed on 10 September 2024).

A Bayesian statistical model was employed to estimate the probability of the homozygous variant’s occurrence in autistic patients, utilizing the following formula according to a previous study [30]:

P(A∣B) = [P(B∣A) × P(A)]/[P(B)];

where P(B∣A) represents the likelihood of a subject with the homozygous mutation being autistic (assumed as 1); P(A) denotes the occurrence of the homozygous variant in 1 subject per 156,777.5 global individuals according to GnomAD database; P(B) represents the probability of a subject having autism spectrum disorder (ASD), estimated as 1 autistic child among 77 healthy children based on previous research carried out in Italy [31].

The Bayesian statistical model was further refined using data from the European (non-Finnish) population in the GnomAD database (accessed on 6 December 2024). This dataset reported 17 European individuals carrying the variant in homozygous conditions out of 1,180,008 subjects. For the Bayesian calculation, this value was normalized to 1 subject per 69,412 individuals.

## 3. Results

### 3.1. Clinical Report

A 4-year-old female was referred to the pediatric neurology clinic for developmental delay and behavioral abnormalities. These concerns were first noticed around 18 months of age. The patient is the first child of healthy, non-consanguineous parents, with an unremarkable perinatal history. Pregnancy and delivery were uneventful, and the patient’s birth weight (3.2 kg), length (50 cm), and head circumference (33.5 cm) were within normal limits. The main clinical features of the examined individual are further detailed in Table 1.

Apgar scores were 9 and 10 at 1 and 5 min, respectively. There were no neonatal complications. Developmental concerns became evident between 15 and 18 months, particularly regarding language delay, poor eye contact, and a lack of social interaction. The patient did not babble as expected and was not using words meaningfully by 18 months. By the age of 2, she exhibited hallmark features of autism spectrum disorder (ASD), including repetitive behaviors (hand-flapping, lining up toys), limited engagement with peers, and hypersensitivity to loud sounds and certain textures (e.g., refusal to wear specific clothing). Behaviorally, she exhibited distress with any changes to routine and demonstrated difficulty with transitions. Notably, the family history for autism was unfavorable. Both the audiogram and impedance test showed a normal range.

### 3.2. Diagnostic Evaluations

The initial diagnostic process began at age 3 with a referral for a comprehensive developmental evaluation. Standardized assessments included the following:

- Mullen Scales of Early Learning (MSEL): At 3 years of age, scores were significantly below average in expressive and receptive language domains (expressive language age equivalent of 18 months, receptive language age equivalent of 21 months). Fine and gross motor skills were slightly delayed but within the lower range of normal (age equivalent 30–33 months).
- Autism Diagnostic Observation Schedule, Second Edition (ADOS-2): The patient had a score within the autism spectrum range with deficits in communication, social interaction, and play behaviors. At the ADOS examination, the patient was placed at Level 2 (phrase speech).
- Vineland Adaptive Behavior Scales, Third Edition (VABS-3): The patient showed delays in adaptive functioning, particularly in the communication and socialization domains. The adaptive behavior composite was significantly below average, with an age equivalent of 2 years in communication and personal–social skills.

Given the severity of symptoms and absence of clear environmental risk factors, the patient was referred for genetic testing. The following tests were performed.

No significant copy number variants (CNVs) were identified upon Chromosomal Microarray Analysis (CMA) and in Fragile X Syndrome testing. whole-exome sequencing (WES) revealed a pathogenic variant in the *ZC2HC1C* gene (c.983dupG).

Additional evaluations included an electroencephalogram (EEG), which gave normal results and no evidence of epileptiform activity. The patient had no history of seizures, but EEG was performed due to the increased risk of epilepsy in patients with monogenic forms of ASD. A brain MRI was performed at the age of 4 years, revealing no anomalies and no structural abnormalities. Metabolic screening was normal and included plasma amino acids, organic urine acids, and serum lactate, which were within normal limits.

### 3.3. Ongoing Management

At the time of diagnosis, the patient was enrolled in an early intervention program with a multidisciplinary approach. She attends a specialized school for children with developmental delays and continues to receive specific therapeutic services, including Speech Therapy, as the patient has acquired a limited spoken vocabulary (~50 words), is primarily nonverbal and uses PECS to communicate basic needs and desires; Occupational Therapy (OT), which addresses sensory processing issues, including tactile defensiveness, and works on improving fine motor skills for activities of daily living (ADLs); sensory integration therapy, provided to help regulate her responses to environmental stimuli. In the last 6 months, she started Applied Behavior Analysis (ABA) (20 h per week) to reduce challenging behaviors (meltdowns, self-stimulatory behaviors) and to promote social skills.

At present, the patient continues to be evaluated by a child psychiatrist for ongoing anxiety and distress related to sensory stimuli. Low-dose Risperidone (0.25 mg/day) has been initiated to manage irritability and aggression, with moderate improvement noted over a 6-month period. Close monitoring for side effects is ongoing, particularly regarding appetite and weight gain. Parental Support and Training has been started and involves behavioral interventions. Parent-mediated social communication programs, such as the Early Start Denver Model (ESDM), have been implemented to support communication at home.

The patient’s progress remains slow but steady. She has shown slight improvements in social engagement and responds better to familiar routines, although language and social deficits persist. The family is currently exploring the possibility of enrolling the patient in clinical trials for targeted therapies related to her specific genetic mutation.

### 3.4. Next Generation Sequencing

**Figure 1:**
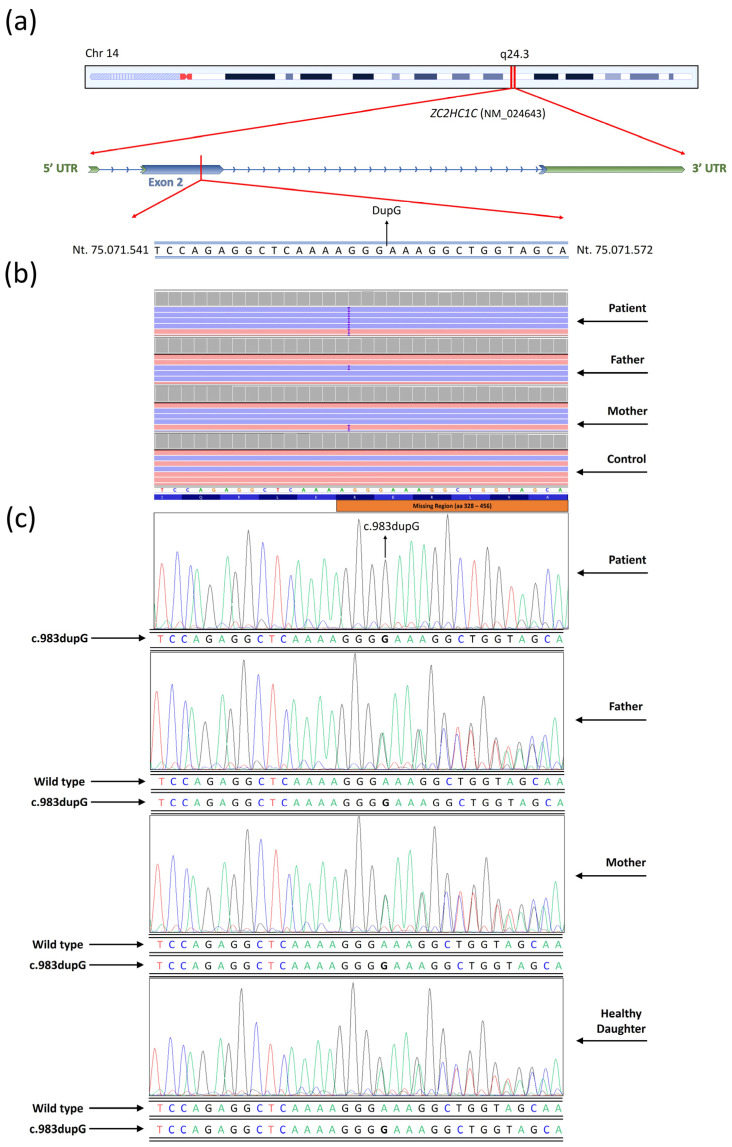
Next-generation sequencing results for observed variants within the *ZC2HC1C* gene. (**a**) Chromosomic and genetic localization of the identified variant. (**a**) was modified from UCSC Genome Browser (http://genome.ucsc.edu). (**b**) Variant detected by the whole-exome sequencing (WES) of the patient, father, and mother (c.983dupG). A health control is also reported (control). Images were obtained by Integrative Genome Viewer (IGV). (**c**) Variant confirmation by conventional Sanger sequencing for the patient affected by autism spectrum disorder (ASD), and both healthy parents (father and mother). Furthermore, the patient’s healthy sister was identified as a heterozygous carrier of the variant.

Whole-exome sequencing (WES) did not identify potential causative variants of unknown genes associated with ASD. Conversely, WES analysis revealed a frameshift insertion variant (c.983dupG) within the *ZC2HC1C* (NM_024643) gene on chromosome 14 (Figure 1).

**Figure 2:**
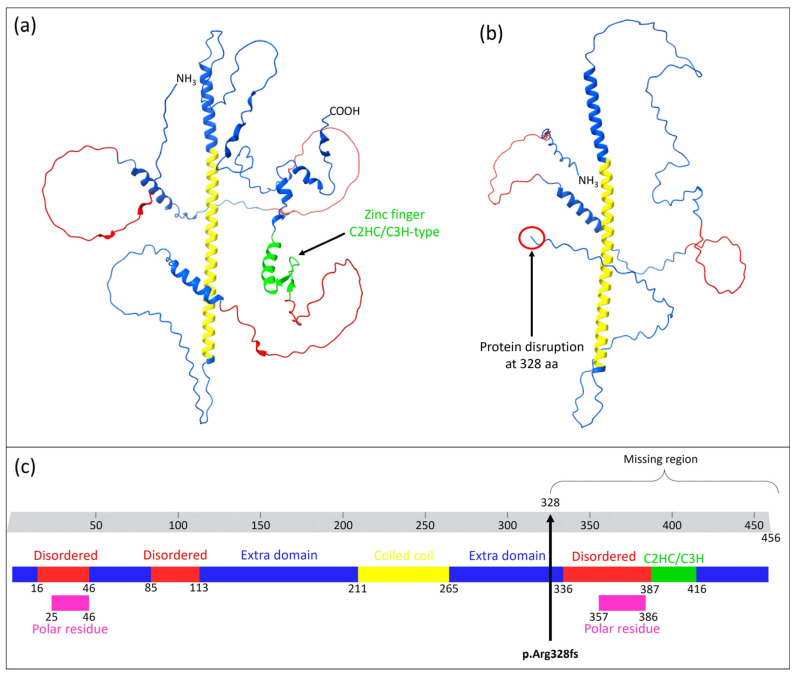
Structure prediction for the wild type (**a**) and mutated (**b**) ZC2HC1C protein. The arrow in (**a**) evidences the zinc finger C2HC/C3H domain. Conversely, the red circle and the arrow reported in (**b**) indicate the specific mutation position on the protein amino acid chain. Proteins structures were generated according to the AlphaFold algorithm. (**c**) Graphical representation of the regions and domain of the ZC2HC1C protein. The arrow represents the mutation p.R328fs that led to the protein disruption. Blue color indicates the extra-domain regions; red color indicates the three disordered regions; yellow color indicates the coiled coil domain; green color represents the atypical C2HC/C3H zinc finger domain; pink color indicates the two polar residues. The Figure was adapted from Uniprot database.

This variant led to the formation of a premature stop codon, resulting in the alteration of the polypeptide chain from the wild type (p.Arg328fs), which consists of 456 amino acids, to the mutated form, which contains only 328 amino acids (Figure 2).

The variant was confirmed through conventional Sanger sequencing (Figure 1c). The observed variant was classified as likely pathogenic by the Varsome algorithm. It potentially disrupts protein domains and regions beyond the amino acid position 328 aa (Figure 1). Specifically affected regions include the disordered region (aa 336 to 388), the polar residue (aa 357 to 386), and the atypical zinc finger C2H2/C3H-type domain (aa 387 to 416) (Figure 1).

### 3.5. Allele Frequency Prediction and Bayes Statistical Probability Model

The allelic frequency documented by the GnomAD database was 0.0025, corresponding to 0.25% among the population (288 individuals out of 113,746). Analysis of Hardy–Weinberg principle revealed that the expected genotypes displaying the observed variant in homozygous conditions were found in 0.36 subjects out of 57,017 individuals in a population (GnomAD) (1 subject per 156,777.5 individuals). The predictive analysis conducted by DOMINO identified this variant as exhibiting autosomal recessive (AR) inheritance patterns. Furthermore, the probability of an Italian patient with ASD having the homozygous variant is 0.049% according to the Bayes statistical model:

P(A∣B) = [P(B∣A) × P(A)]/[P(B)];

P(A∣B) = [1 1/156,777.5]/[1/77];

P(A∣B) = 77/156,777.5 = 0.0004908

The Bayesian statistical model was further refined for the European (non-Finnish) population belonging to the same ethnic group of the individual examined in this study. Specifically, based on observed homozygous genotypes, 1 in 69,412 individuals displayed the variant in a homozygous condition. Within this context, the following formula was applied to determine the probability of a European individual with the variant in a homozygous condition being autistic.

P(A∣B) = [1 1/69,412]/[1/77];

P(A∣B) = 77/69,412 = 0.0011093

## 4. Discussion

### 4.1. Patient’s Phenotype and Variant Identification

The clinical case examined in the current study displays severe autism spectrum disorder (ASD) conditions. WES unveiled the presence of a homozygous insertion located in the zinc finger gene *ZC2HC1C*. In particular, WES did not identify any variants in known genes associated with the patient’s condition. However, the possibility that other genetic variants in regions not covered by WES could contribute to the patient’s phenotype cannot be excluded. Additionally, we noted that this gene is not listed in the SFARI or AutDB databases, both of which are key resources for autism research. This highlights that our findings represent the first, albeit modest, association of this gene with autism. The variant was found in the heterozygous condition in the healthy patient’s sister. It is worth noting that the variant was already reported in the dbSNPS database with the entry code rs528262343, but no clinical details have been indicated. Numerous researchers have emphasized the role of dysfunctional zinc finger proteins in the onset of neurodevelopmental disorders, including autism [1]. To date, *ZC2HC1C* shows no MIM phenotype number associated with a specific phenotype. Additionally, disease-causing mutations were not found in the clinical phenotype databases (ClinVar, HGMD, LOVD). In silico predictive analysis indicates the likely pathogenic significance of the observed variant. The identified insertion entailed a duplication of G within exon 2 of *ZC2HC1C* on chromosome 14 at position 983. In this manuscript, we propose that the detected mutation led to a premature stop codon at amino acid 328 within the ZC2HC1C protein, resulting in the removal of three functional domains: the disordered region, the polar residues’ compositional bias, and the zinc finger C2HC/C3H type (Figure 2). Notably, the protein organization of ZC2HC1C diverges from that of conventional zinc finger proteins, which typically feature C2HC domains. Specifically, ZC2H1C lacks the classic zinc finger domain but instead displays the C2HC/C3H type. It is noteworthy that this domain has limited references available in the literature.

Furthermore, the protein structure prediction analysis revealed a loss of 52 hydrogen bonds from the wild-type protein to the mutated protein. Notably, it is well established that variations in hydrogen bonds can significantly impact protein folding and its tertiary structure, exerting a negative effect on the protein function [32,33,34].

Remarkably, we are implementing findings from the literature by hypothesizing, for the first time, a plausible correlation between *ZC2HC1C* and autism. According to the data obtained from The Human Protein Atlas database, the gene is primarily expressed in the testicles. However, its secondary expression is observed in brain tissues, with the highest expression noted in the choroid plexus and midbrain. Interestingly, as documented [35], the choroid plexus (ChP) produces protein-rich cerebrospinal fluid and matures during gestation, prior to brain development. It is assumed that ChP dysfunction has a profound effect on developmental neuropsychiatric disorders, such as Autism Spectrum Disorder (ASD). Furthermore, it was emphasized that autistic behavior arises from a dysfunctional midbrain dopaminergic system [36].

### 4.2. Allele Frequency and Bayes Statistical Model

The variant c.983dupG of the gene *ZC2HC1C* shows an allelic frequency of 0.25% in the worldwide population. It is important to note that the allele frequency reported in the GnomAD database does not account for the phenotypes of individuals carrying the variant. The Hardy–Weinberg principle, based on data from GnomAD, suggests homozygous occurrence in 1 subject per 156,777.5 individuals. Hypothesizing autosomal recessive inheritance (supported by DOMINO prediction and the presence of the variant in the heterozygous condition in the patient’s healthy parents and sister), and considering that approximately 1.3% of children aged 7–9 years in Italy are affected by ASD [31], we indicate a probability of 0.049% of a patient with ASD having the homozygous variant c.983dupG within the *ZC2HC1C* gene, according to the Bayes statistical model. For this model, we considered the incidence of autism in Italy, referring to the previously mentioned study [31] for several reasons. First, this assessment is highly reliable as it is based on certified ASD diagnoses obtained from the Ministry of Education’s records. Furthermore, the sample used in our study is representative of the Italian population, as it includes students from the North, Center, and South of the country. Lastly, the study by Scattoni et al. was recently conducted and is reliable, being directly funded by the Italian Ministry of Health. The Bayesian statistical model was further refined using data from the European population in the GnomAD database. Notably, the probability of a European individual carrying the homozygous variant identified in this study exhibiting ASD was calculated to be 0.11%. However, it is important to note that this calculation should be further refined for the Sicilian population, which is documented to exhibit high genetic heterogeneity [37,38].

### 4.3. Protein–Protein Interaction Prediction

**Figure 3:**
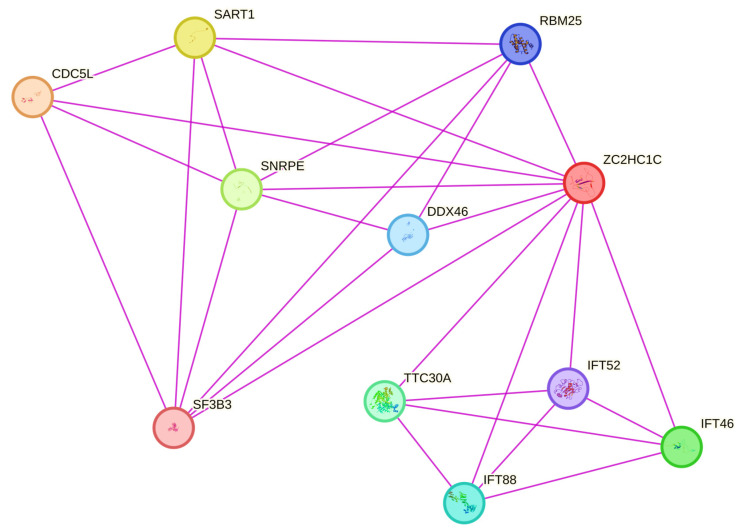
Graphical representation of the interactions observed in STRING database involving ZC2HC1C protein (STRING Node ID: 9606.ENSP00000435550). The respective interaction score for each node assigned by the STRING database is reported in Table 2.

According to various protein–protein interaction databases, *ZC2HC1C* interacts with several genes crucial for primary cilia development. It is widely acknowledged that disturbances in this mechanism can lead to neurodevelopmental disorders, including ASD [21,22]. Protein–protein interactions were analyzed using the STRING, BioGRID, and IntAct databases. The BioGRID database highlighted a significant number of high-throughput physical interactions for ZC2HC1C, including 112 interactions and 102 unique interactors. Notably, the interaction scores between ZC2HC1C and other proteins were as follows: TTC30A (0.9933), IFT46 (0.9991), CUL7 (1), SIKE1 (1), and FBXW8 (0.999). CUL7 and FBXW8 are key components of the ubiquitin–proteasome system (UPS), and both have been shown to form complexes involved in placental development [39]. As documented, defects in this system result in postnatal growth retardation in mice [40]. Within the context of intraflagellar transport (IFT), ZC2HC1C was also found to interact with IFT46 (0.9991), IFT52 (0.9974), IFT88 (0.9952), IFT57 (0.9793), IFT74 (0.9674), IFT172 (0.9612), IFT81 (0.9092), and IFT22 (0.7731), as annotated in the BioGRID database. Furthermore, interactions with MAN2C1 (0.9972), GPHN (0.9727), LRP2 (0.9711), and HSPA9 (0.824) are noteworthy. These proteins are all listed in the OMIM database with specific pathogenic phenotypes and exhibit autosomal recessive inheritance patterns linked to neurological disorders. For example, MAN2C1 is associated with congenital disorder of deglycosylation 2 (MIM #619775), GPHN with molybdenum cofactor deficiency C (MIM #615501), LRP2 with Donnai–Barrow syndrome (MIM #222448), and HSPA9 with Even-plus syndrome (MIM #616854). While autistic features are not explicitly noted in the clinical descriptions of these syndromes, they do include various neurological and psychiatric disorders. Furthermore, the interactions observed in the STRING database are depicted in Figure 3 and listed in Table 2 with the respective interactions scores.

It is worth noting that several interactions annotated in the STRING database involved other organisms, including *Saccharomyces cerevisiae* and *Schizosaccharomyces pombe*.

As previously indicated, a moderated protein–protein interaction was observed with a wide spectrum of ciliary tip proteins involved in intraflagellar trasport, as reported in an interactome study [17]. In particular, TTC30A, IFT46 and HOOK2 play a pivotal role in ciliogenesis, orchestrating anterograde intraflagellar transport (IFT) [18,46,47,48,49,50,51,52]. HOOK2 is a centrosome fusion protein operating as a dynein adaptor and microtubule-binding protein [53]. In fact, ZC2HC1C has been identified as a novel ciliary tip protein localized in the cilia of hTERT-RPE1 cells [54]. It co-transports with intraflagellar transport (IFT) trains along the axoneme, with IFT74 showing higher mobility. Notably, some stationary IFT74 positions overlap with ZC2HC1C, suggesting its role in ciliary dynamics and IFT interactions. In addition, as previously documented, elevated mRNA levels of *ZC2HC1C* were found in mouse cilia, suggesting its potential role in ciliary function [11,12].

Cilia are microtubule-based structures that project into the extracellular space. As is well known, dysfunction of the primary cilia contributes to the pathophysiology of several brain malformations, intellectual disabilities, epilepsy, psychiatric disorder and autism [15,22,55,56]. Dysfunctions in this process lead to the onset of heterogeneous phenotypes, named as ciliopathies [57]. Primary cilia are essential organelles in neuronal development, acting as signaling hubs that regulate key pathways such as Sonic Hedgehog (Shh). This pathway influence neurogenesis, cell differentiation, and the formation of neural circuits [58,59]. In addition, mutations in zinc finger proteins have often been associated with autism and ciliopathies [60,61,62]. As has been documented, ciliogenesis is often linked to both psychiatric and neurodevelopmental disorders, encompassing autism, highlighting its critical role in brain development and function [19,20,21,22]. Reinforcing the association with cilia development, ZC2HC1C was found to be localized in the ciliary tip region [17]. Notably, we propose a plausible association between ZC2HC1C and primary cilia formation as a result of its interaction with a diverse array of IFT proteins. However, ZC2HC1C remains largely unknown as it has not been assigned any KEGG orthology (KO) code, primarily because of the gene’s unknown function. With regard to its biological function, as annotated in the QuickGO database, ZC2CH1C enables protein binding (GO:0005515) and metal ion binding (GO:0046872).

### 4.4. Further Analysis

Functional studies are required to validate the pathogenic and causative effect of the ZC2H1C gene in ASD. Furthermore, studies involving autistic individuals are needed to confirm the occurrence of the homozygous variant (0.049%) c.983dupG within the ZC2HC1C gene in Italian patients showing ASD. We finally propose adding this gene to the list of susceptibility genes for autism. Finally, the predicted protein–protein interaction should be validated using co-immunoprecipitation assays for demonstrating the proposed role of ZC2HC1C in ciliogenesis.

## 5. Conclusions

In this manuscript, we establish a probable association between the homozygous variant identified within the *ZC2HC1C* gene and autism spectrum disorder (ASD). Currently, the function of ZC2HC1C remains poorly understood; in fact, no OMIM code has been assigned. Although predominantly expressed in testis, this gene also shows expression in brain tissues previously associated with ASD. The predictive analysis classified the identified variant as likely pathogenic due to the loss of various functional domains including the zinc finger C2HC/C3H type. Furthermore, estimation of the homozygous variant’s frequency in the population, along with the Bayes probability model, supports our hypothesis of the low prevalence of this rare variant among ASD subjects. Functional analyses are required to confirm *ZC2HC1C* as a susceptibility gene for ASD.

## Figures and Tables

**Table 1:** Main clinical features of the examined individual, across different clinical stages.

Clinical Features	
Family	Non-consanguineous healthy parents
Sex	Female
Pregnancy history	Uneventful
Delivery	Vaginal at term
Growth (at birth—40 weeks)	
Weight	3630 gr
Lenght	50 cm
Occipitofrontal circumference	33.5 cm
Growth	
Height	108 cm (25° centile)
Weight	19 kg (50° centile)
Occipitofrontal circumference	50 cm (25° centile)
Craniofacial anomalies	Brachicephaly, short neck, epicanthus, wide nasal bridge, flat philtrum, prognatism, large anteverted ears, brachidactily, brachycephaly
Other anomalies	Tireoglossus duct cist, supranumerary nipple
Development	Global developmental delay, iq (scale) 82, normal gross motor skills, normal fine motor skills, language delay, autism spectrum disorder, aggressive behavior/self-injurious behavior, stereotypic behavior, hyperactivity
Neurological features	Normal muscle tone, limb hypertonia, tetraplegia, no seizures, no EEG abnormalities

**Table 2:** List of interactions involving the ZC2HC1C protein (STRING Node ID: 9606.ENSP00000435550) as Node 1, including the first 10 interacting proteins (Node 2) with a STRING score greater than 0.5.

Node 2	Annotation	Score	Organism	Reference
CDC5L	Cell division cycle 5-like protein	0.786	*S. pombe*	[41]
SART1	U4/U6.U5 tri-snRNP-associated protein 1	0.638	*S. cerevisiae*	[42]
SNRPE	Small nuclear ribonucleoprotein E	0.62	*S. cerevisiae*	[43]
IFT46	Intraflagellar transport protein 46 homolog	0.606	*H. sapiens*	[17]
TTC30A	Intraflagellar transport protein 88 homolog	0.606	*H. sapiens*	[17]
IFT88	Intraflagellar transport protein 88 homolog	0.594	*H. sapiens*	[17]
DDX46	Probable ATP-dependent RNA helicase DDX46	0.584	*S. cerevisiae*	[44]
RBM25	RNA-binding protein 25	0.564	*S. cerevisiae*	[45]
IFT52	Intraflagellar transport protein 52 homolog	0.551	*H. sapiens*	[17]
SF3B3	Splicing factor 3B subunit 3	0.526	*S. cerevisiae*	[45]

## Data Availability

The data presented in this study are in the main text. Further data are available on request from the corresponding author.

## Abbreviations

The following abbreviations are used in this manuscript:

ASD	Autism spectrum disorder
AR	Autosomal recessive
CNVs	Copy number variants
EEG	Electroencephalogram
ESDM	Early Start Denver Model
ITF	Intraflagellar transport
NGS	Next-generation sequencing
WES	Whole-exome sequencing
